# 2021 Nobel Prize for mechanosensory transduction

**DOI:** 10.1007/s12551-022-00935-9

**Published:** 2022-02-19

**Authors:** Boris Martinac

**Affiliations:** Molecular Cardiology and Biophysics Division, Victor Chang Cardiac Research Institute and St. Vincent’s Medical School, Darlinghurst, NSW 2010 Australia

**Keywords:** Mechanosensitive ion channels, MscL, MscS, Piezo1, Piezo2

## Abstract

Written by someone who has worked in the mechanobiology field for close to 40 years, this commentary describes some historical background to the recent award of one-half of the Nobel Prize for Physiology or Medicine to Ardem Patapoutian for his discovery of the family of mechanosensitive Piezo ion channels, which function as mechanoreceptors feeling the environment in senses such as touch, pain, and proprioception.

The shared Nobel Prize for Physiology or Medicine 2021 awarded to David Julius and Ardem Patapoutian for their respective breakthrough discovery of the vertebrate thermo-sensory TRP and mechanosensory Piezo ion channels recognizes the significance of the evolutionarily inherent ability that living organisms, from bacteria to humans, possess in sensing and responding to changes in their surrounding environment. This commentary is restricted to the prize relating to mechanosensitive ion channels.

Given the essential role of water for the existence of life and the presence of osmotic forces throughout the evolution of different life forms on Earth, mechanosensitive ion channels may be the oldest type of mechanoreceptors that evolved as primary signaling molecules supporting mechanosensory physiology of living organisms. Without the ability to perceive sensations of touch, hearing, sight, taste, smell, temperature, or pain, the outside world would cease to exist for vertebrate organisms, including humans, which emphasizes the importance of sensory input for the existence of life. To this point, Piezo2 members of the Piezo ion channel family serve as mechanoreceptors feeling the environment in senses such as touch, pain, and proprioception (Ernfors et al. 2021) (Fig. 1).

**Fig. 1:**
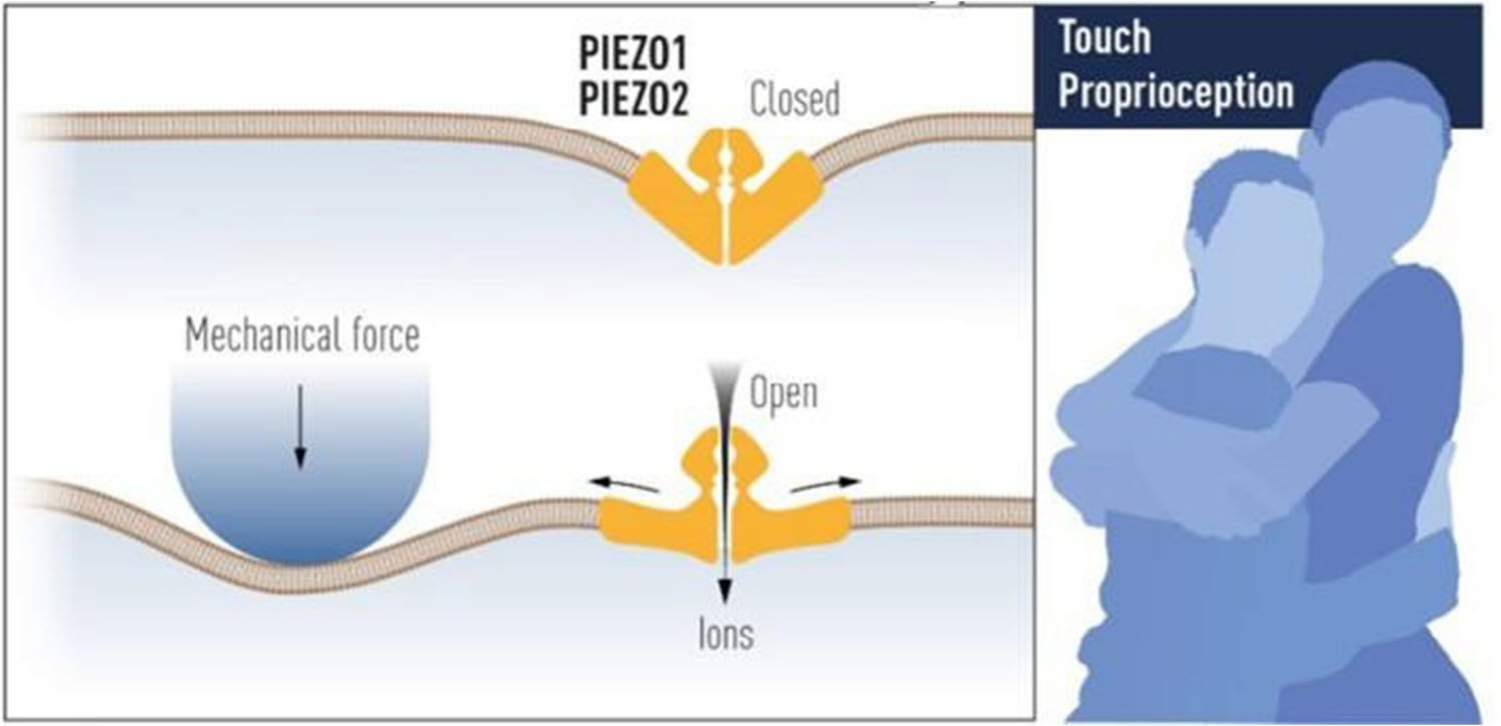
Piezo family of ion channels. Piezo1 and Piezo2 are members of a new family of non-selective cation channels permeable to Ca^2 +^ that are widely expressed in tissues of the hollow organs (lung, stomach, bladder, intestines) where they function as molecular mechanosensors. Cryo-electronmicroscopy structure of both channels revealed their curved structure in the membrane, which when flattened by mechanical force stretching the membrane, opens the channel pore (left panel). A hug is an example of the sense of touch in which Piezo2 functions as the mechanoreceptor (right panel) (Adopted and reproduced from (Ernfors et al. 2021) with permission)

## Brief history

**Fig. 2:**
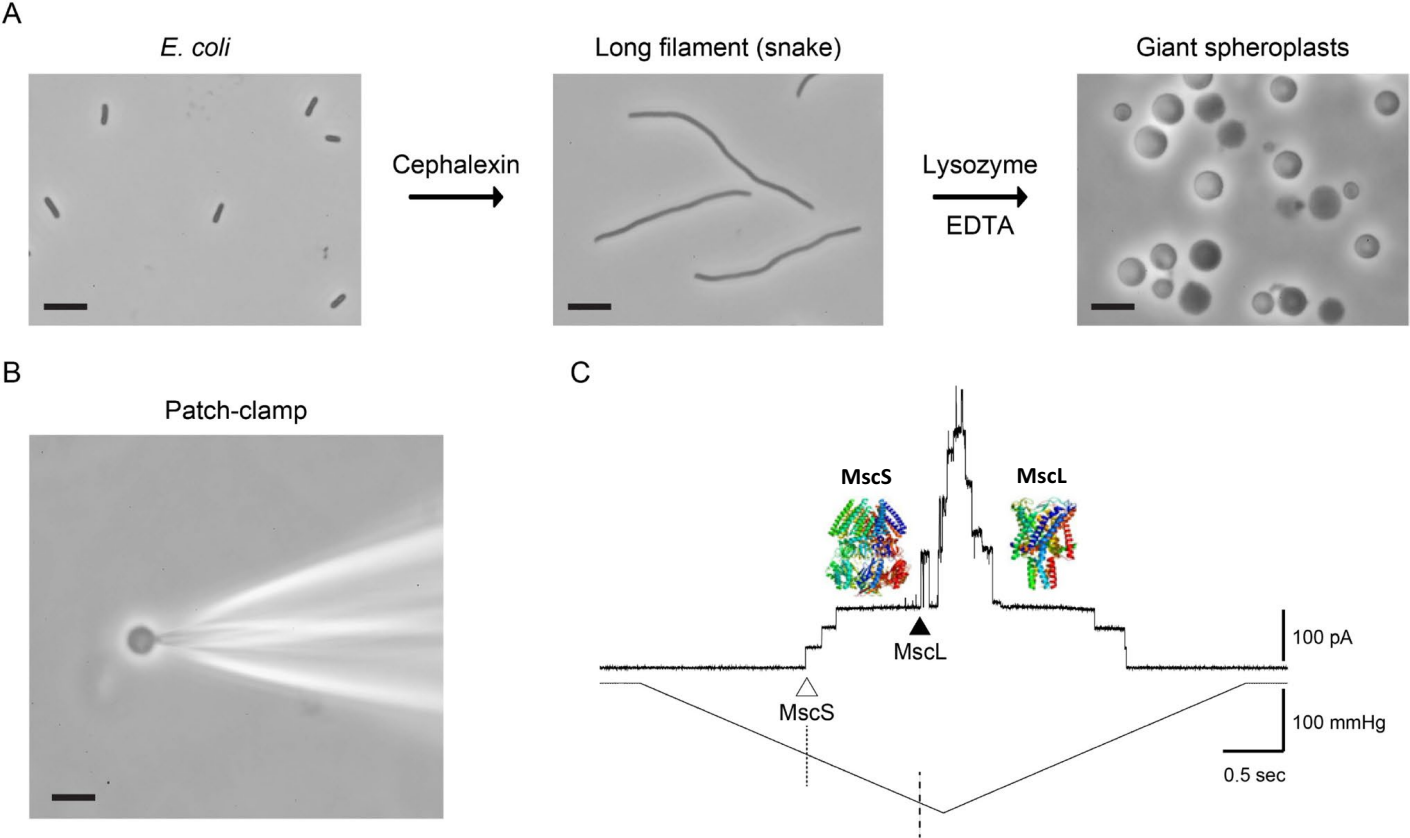
Preparation of and patch-clamp recording from E. coli giant spheroplasts. The major steps during generation of giant spheroplasts from E. coli (scale bar corresponds to 5 μm in all images) including generation of “snakes” and converting them into giant spheroplasts amenable to the patch clamp recording (top row). A phase contrast microphotograph of the patch pipette with a giant spheroplast at the tip (bottom row left). Activity of MscS and MscL channels of small and large conductance, respectively, recorded from an inside-out giant spheroplast patch (bottom row right). The upper trace shows the current traces of single MscS (open triangle) and MscL (filled triangle) channels opening upon application of negative pressure ramp (suction depicted by lower trace) applied to a patch pipette using a high-speed pressure clamp device. MscS activation threshold (− 114.5 mmHg) is indicated by the dotted line, whereas the activation threshold of MscL (− 195.2 mmHg) is indicated by the dashed line in good agreement with the previously reported results (Nomura et al. 2012; Yoshimura et al. 1999). Both MscS and MscL function as safety valves protecting bacteria from a sudden rise in cellular turgor pressure due to a hypoosmotic shock (Levina et al. 1999). Pipette potential was held at + 30 mV (Reproduced with permission from (Martinac et al. 2013))

The existence of mechanosensitive ion channels was first postulated in 1950 by Bernard Katz, who won the Nobel Prize for Physiology or Medicine in 1970 for his work on nerve physiology (Katz 1950). A quarter of a century later, Georg von Békésy, the 1961 Nobel Prize winner for Physiology or Medicine, discussed a possible existence of a mechanical receptor in frequency discrimination in the ear (Von Bekesy 1974). Several years later, Corey and Hudspeth suggested the existence of vertebrate mechanosensitive ion channels in bullfrog cochlear hair cells (Corey and Hudspeth 1979). Shortly after, thanks to the advent of the patch clamp technique (Hamill et al. 1981), the first single mechanosensitive ion channels were recorded from chick skeletal muscle by Guharay and Sachs (Guharay and Sachs 1984), from innervated muscle of Xenopus laevis by Brehm and collagues (Brehm et al. 1984) and from giant spheroplasts of E. coli by Martinac and colleagues (Martinac et al. 1987). It is to the credit of the Nobel Assembly, who duly recognized the pioneering studies on bacterial mechanosensitive channels (Fig. 2), which firmly established the existence of mechanosensitive channels in the late 1980s (Cox et al. 2018; Delcour et al. 1989; Martinac et al. 1987; Sukharev et al. 1994). This collective work ultimately paved the way to this year’s Nobel Prize for the discovery of the Piezo mechanoreceptor family (Ernfors et al. 2021).

## Discovery of Piezo1 and Piezo2

**Fig. 3:**
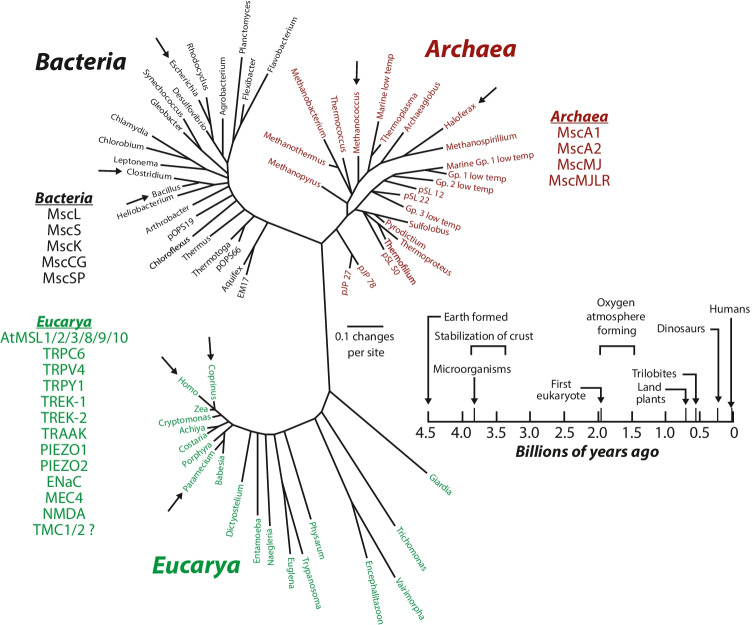
Universal phylogenetic tree. Life on Earth is organized in three kingdoms of living organisms based on small subunit tRNA sequences (Pace 1997). Examples of the mechanosensitive channels that have been identified and characterized in each kingdom are listed next to the group of organisms in which they are found (arrows). Biological timescale from the time of the Earth’s formation 4.6 billion years ago to the time of human origin (bottom right) (Woese 1994). The oldest microfossils of prokaryotic cells are approximately 3.5 billion years old (Reproduced with permission from (Martinac and Cox 2017))

Following on these initial discoveries, numerous mechanosensitive channels were functionally identified in cells and organisms from all three domains of life, Bacteria, Archaea, and Eukarya (Fig. 3) (Martinac and Cox 2017). The sheer number and occurrence of mechanosensitive channels eventually cast doubt on their biological reality and relevance, so that at some point they were suspected to be nothing more than artifacts of the patch-clamp recording technique. Their existence was finally put beyond doubt with the isolation, cloning, and determination of the 3D X-ray crystal structure of MscL, the bacterial mechanosensitive channel of large conductance from Mycobacterium tuberculosis, as the first channel of this kind identified at a molecular level (Chang et al. 1998; Hamill and Martinac 2001; Kefauver et al. 2020; Sukharev et al. 1994). There are now many examples of mechanosensitive channels that have been cloned in all types of organisms (Martinac and Cox 2017), and over the last 20 odd years, the information on their molecular identity and structure has become available.

**Fig. 4:**
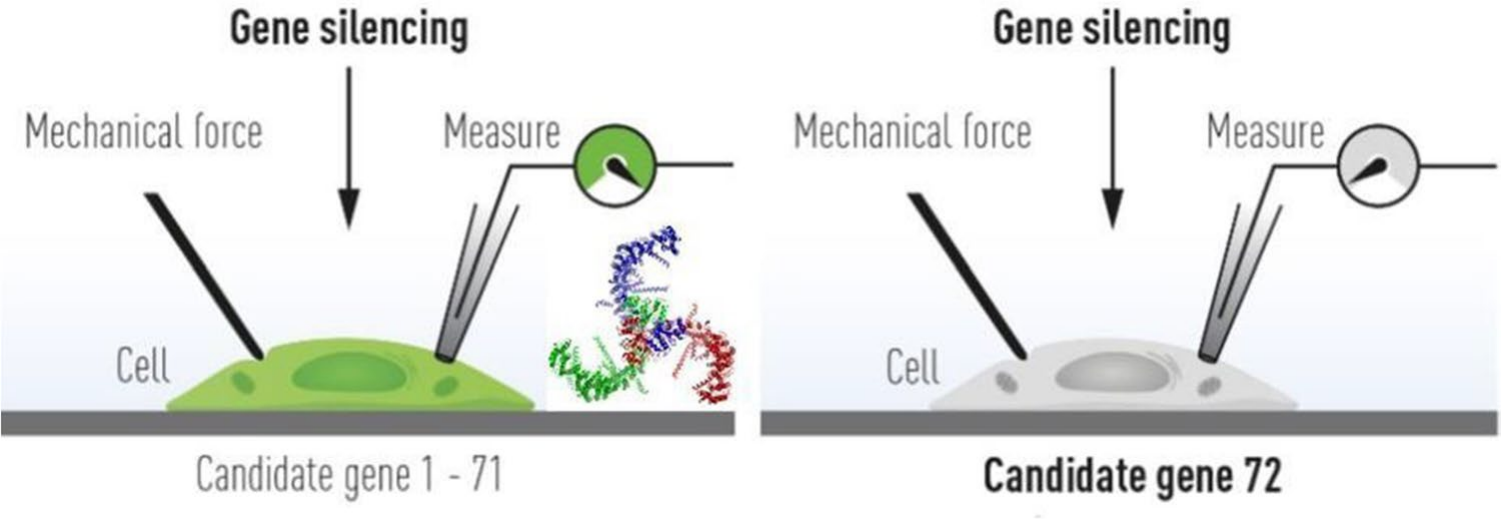
The discovery of Piezo channels. Piezo ion channels were discovered by using gene silencing of 72 candidate genes in the mechanosensitive mouse cell line Neuro2a and searching for a loss of mechanoreception. This enabled discovery of the Piezo ion channel family. Cryo-EM structure of the mouse Piezo1 triskelion with propeller-like arms composed of transmembrane helices forcing the membrane to curve (inset) (Reproduced and modified from (Ernfors et al. 2021) with permission)

The mammalian Piezo mechanosensitive channel family consisting of the Piezo1 and Piezo2 members was discovered just over a decade ago (Fig. 4) (Coste et al. 2010, 2012). Since their discovery, both channels have been extensively studied in laboratories all around the world. Both Piezo1 and Piezo2 are non-selective cationic channels permeable to Ca^2+^, whose gating can be stimulated by a variety of mechanical stimuli, including membrane stretching, compression, poking, and shear stress (Kefauver et al. 2020; Martinac and Cox 2017). Rapid growth in Piezo channel research very quickly led to determination of the 3D molecular structure of both Piezo1 (Guo and MacKinnon 2017) (Saotome et al. 2018; Zhao et al. 2018) and Piezo 2 (Wang et al. 2019) showing that both channels are homotrimers formed as a triskelion (viewed from the top) (Fig. 4, inset) (Guo and MacKinnon 2017). Like bacterial MscL and MscS channels (Cranfield et al. 2018), Piezo channels are also inherently mechanosensitive by sensing mechanical force directly through the lipid membrane (Cox et al. 2016; Syeda et al. 2016). However, although Piezo1 channel is gating according to the force-from-lipids paradigm (Martinac et al. 1990; Kung 2005; Teng et al. 2015), it has been shown that extracellular matrix and cytoskeleton can markedly modulate mechanical stimuli detected by this ion channel (Cox et al. 2019).

The significance of the Piezo channel discovery comes also from their implication in a number of mechanopathologies linked to malfunctioning of these ion channels in humans. Hereditary Xerocytosis, a familial anemia, is caused by a gain-of-function mutation in Piezo1 characterized by slowed inactivation resulting from three missense mutations and one recurrent duplication in the Piezo1 gene, with all mutations located at the C-terminal half of Piezo1 (Albuisson et al. 2013). Generalized lymphatic dysplasia, which also manifests itself in varying degrees of anemia in addition to defects in lymphatic development, results from loss-of-function Piezo1 mutations characterized by reduced sensitivity to the applied mechanical force (Fotiou et al. 2015). Furthermore, given that carcinogenesis is associated with significant changes in mechanical properties of the affected cells and tissues that contribute to the malignancy of the respective types of cancers, the expression of both Piezo1 and Piezo2 varies largely in different types of cancers (De Felice and Alaimo 2020).

The 2021 Nobel Prize awarded for discovery of the Piezo1 and Piezo2 mechanoreceptors marks a great year for the mechanobiology field by recognizing the essential role that mechanical force, in all its manifestations, plays for living organisms. With regard to Piezo channels, the Prize embodies the significant role these mechanoreceptors play in a variety of physiological processes in vertebrates. It also recognizes the potential of both channels for the development of novel treatments for a wide range of mechano-channelopathies in the future. This shines a spotlight on the role mechanoreceptors play in the sustainability of different life forms, in a variety of environments, including microgravity for human colonization of outer space (Basirun et al. 2021). This year’s Nobel Prize for discovery of the Piezo channel family provides significant impetus for research in mechanotransduction.
